# Manifestation of Spontaneous Rupture of the Urinary Bladder in Pregnancy: A Systematic Review of the Literature

**DOI:** 10.7759/cureus.44643

**Published:** 2023-09-04

**Authors:** Amene Ranjbar, Vahid Mehrnoush, Farideh Montazeri, Fatemeh Darsareh, Walid Shahrour, Nasibeh Roozbeh, Mojdeh Banaei, Mozhgan Saffari

**Affiliations:** 1 Fertility and Infertility Research Center, Hormozgan University of Medical Sciences, Bandar Abbas, IRN; 2 Department of Urology, Northern Ontario School of Medicine, Thunder Bay, CAN; 3 Mother and Child Welfare Research Center, Hormozgan University of Medical Sciences, Bandar Abbas, IRN

**Keywords:** spontaneous rupture of urinary bladder, systematic review, pregnancy, urinary bladder rupture, spontaneous bladder rupture

## Abstract

Spontaneous rupture of the urinary bladder (SRUB) during pregnancy is a potentially fatal event that necessitates immediate surgery. The aim of this systematic review is to determine the symptoms, causes, associated factors, and prognosis of SRUB in pregnancy. We searched the literature from inception until December 2022 using the Cochrane Central Register, PubMed, EMBASE, ProQuest, Scopus, and Google Scholar. Articles not in English and those unrelated to the topic were excluded. The JBI Critical Appraisal Checklist for case reports was employed for the risk of bias assessment. The search strategy yielded 312 citations; 29 full-text articles were evaluated for eligibility after screening. Five case reports were included in this review. The age range of the cases was 27-39 years. Four cases were in their second trimester of pregnancy, and one was in her third. Two cases had undergone previous cesarean sections, and one had an appendectomy and salpingectomy due to an ectopic pregnancy. One case reported a history of alcohol and drug abuse. The most common symptoms were abdominal pain, abdominal distension, oliguria, voiding difficulty, hematuria, fever, and vomiting. The diagnosis of SRUB was primarily made via emergency laparotomy due to the patients' critical conditions. Beyond its diagnostic role, laparotomy was also the treatment method in all cases. Tear repair, antibiotic therapy, and urinary catheterization were all integral parts of the treatment. Four cases resulted in an uneventful pregnancy and a healthy, full-term baby. In one case, a hysterectomy was performed due to a combined uterus and bladder rupture. SRUB often presents with non-specific symptoms, leading to a delayed diagnosis. A high index of suspicion is essential when a pregnant woman exhibits urinary symptoms and severe abdominal pain, especially in those with a history of previous surgery. Early detection and treatment of SRUB are critical for an uneventful recovery.

## Introduction and background

The incidence of spontaneous rupture of the urinary bladder (SRUB) is about 1 in every 126,000 people [1]. Although the cause of SRUB is unknown, a predisposing factor makes a patient susceptible to bladder rupture [2]. In general, SRUB can be explained by pathological bladder rupture [3] and idiopathic bladder rupture [4]. The most common cause of pathological bladder rupture is lower urinary tract obstruction, bladder tumor or inflammation, pregnancy-related causes, bladder dysfunction, pelvic radiotherapy, and a history of bladder surgery or bladder diverticulum [5]. Spontaneous idiopathic bladder rupture is uncommon and, as the name suggests, has no identifiable underlying cause. Early detection of SRUB is challenging, and there needs to be published guidelines for management [6]. SRUB during pregnancy, childbirth, and postpartum is a potentially fatal event that necessitates immediate surgery to minimize morbidity and mortality. Although most SRUB has been reported after childbirth [7,8], studies on bladder rupture during pregnancy are limited in the literature [9-13], making it challenging to fully comprehend the corresponding underlying causes. This systematic review aims to identify SRUB's manifestation, causes, associated factors, and prognosis during pregnancy.

## Review

This protocol was designed based on the Preferred Reporting Items for Systematic Review and Meta-Analysis Protocols [14]. The protocol for this review was submitted at PROSPERO on 20 March 2022 with I.D. number CRD42022319511. It should be noted that the protocol was also published previously [15]. Because the study only included previously published studies, no ethical approval was required. Patients or the public were not involved in this research.

Objective of the study

The objective of the study was to determine the manifestation, causes, related factors, and prognosis of SRUB in pregnancy.

Review questions

The review questions were as follows: (1) What is the manifestation of SRUB during pregnancy?; (2) Are there any predisposing factors that can aid in predicting the diagnosis of SRUB in pregnant women?; (3) What is the best method of diagnosis and treatment of SRUB during pregnancy?; and (4) What is the prognosis of SRUB in pregnancy?

All studies representing pregnant women with SRUB at any trimester of pregnancy were reviewed. Non-English articles, as well as those unrelated to the topic, were excluded. Letters to the editor and reviews were also excluded. The search strategy targeted studies published from inception to December 2022. Databases searched included the Cochrane Central Register, PubMed, EMBASE (Via Ovid), ProQuest, Scopus, and Google Scholar. Keywords were selected based on the MeSH terms and included "bladder rupture,"; "spontaneous bladder rupture,"; "SRUB,"; "urinary bladder rupture,"; "rupture of the urinary bladder,"; "ruptured urinary bladder,"; "rupture of bladder"; "pregnancy"; "gestation"; "pregnant women"; "birth"; "childbirth"; "parturition"; "gravidity"; and "parity." These keywords were combined using Boolean "OR" and "AND" operators. In addition, the reference lists of the identified articles were also searched along with hand-searching to ensure that all documents were retrieved, which were combined using Boolean "OR" and "AND" operators. Additional file 2 provides the details of the search strategy. An experienced researcher, Amene Ranjbar, conducted all database searches. After removing duplicates, two researchers, Farideh Montazeri and Mozhgan Saffari, independently screened the titles, abstracts, and full texts of potential studies based on pre-defined eligibility criteria. Any disagreements were resolved through consensus or consultation with a senior researcher, Amene Ranjbar. The study selection process was documented using a PRISMA flow diagram. Two investigators (Farideh Montazeri and Mozhgan Saffari) independently extracted data. Vahid Mehrnoush solved disagreements. The following items were extracted: type of study, population characteristics, method of diagnosis, risk factors, type of treatment, and prognosis. The methodological quality of the included studies was assessed using the JBI Critical Appraisal Checklist for case reports [16].

Results

**Figure 1:**
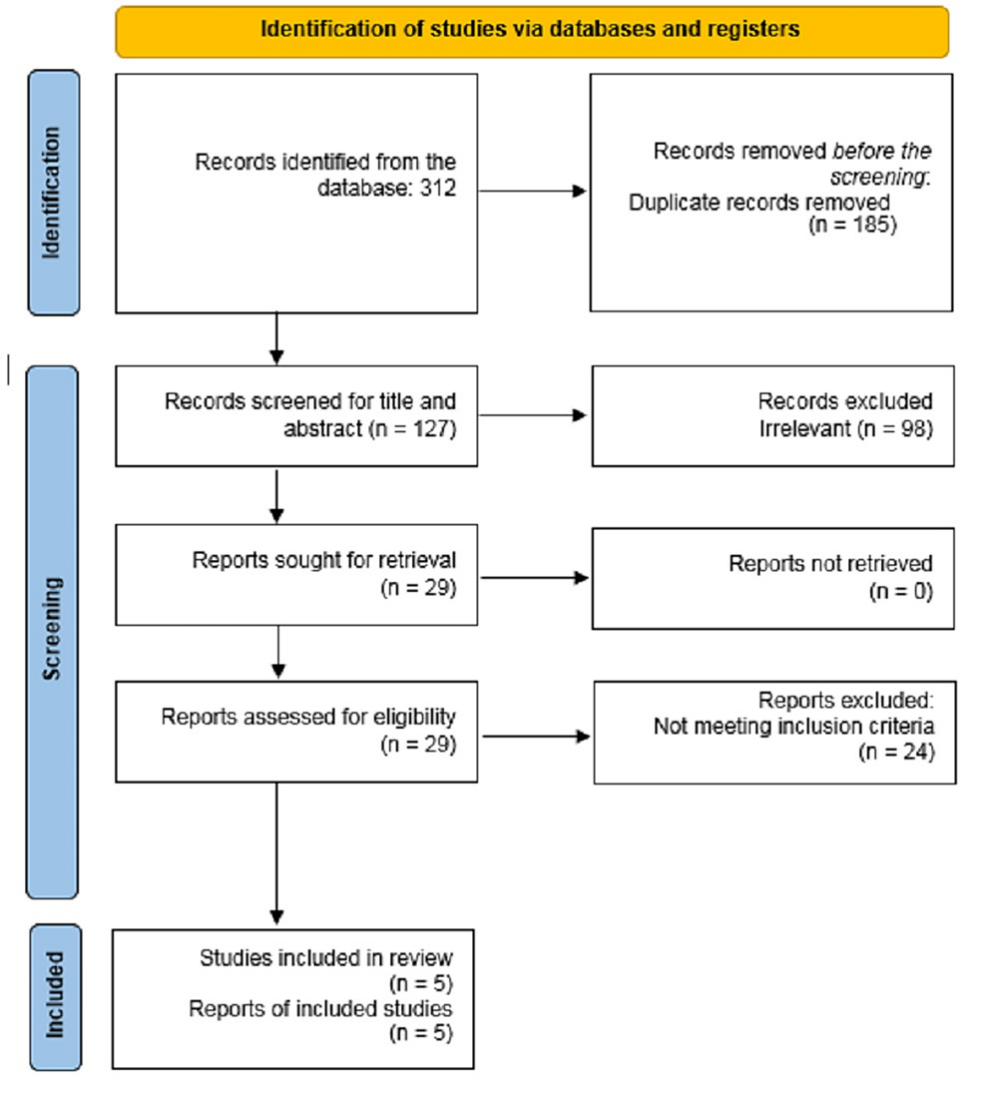
Flowchart of the study.

The search strategy yielded 312 citations. After removing duplicates and completing the screening, 29 full-text articles were evaluated for eligibility (as shown in the PRISMA Flow Diagram, Figure 1).

Five case reports were included in this review. The critical appraisal checklist of included studies is shown in Table 1.

**Table 1:** JBI Critical Appraisal Checklist for Case Reports. D1: Were the patient’s demographic characteristics clearly described? D2: Was the patient’s history clearly described and presented as a timeline? D3: Was the patient's current clinical condition on presentation clearly described? D4: Were diagnostic tests or assessment methods and the results clearly described? D5: Was the intervention(s) or treatment procedure(s) clearly described? D6: Was the post-intervention clinical condition clearly described? D7: Were adverse events (harms) or unanticipated events identified and described? D8: Does the case report provide takeaway lessons?

Title	Reference	D1	D2	D3	D4	D5	D6	D7	D8	Overall Appraisal
Spontaneous rupture of the urinary bladder in the third trimester of pregnancy	[9]	Yes	Yes	Yes	Yes	Yes	Yes	Yes	Yes	Included
Spontaneous rupture of bladder due to a prolapsed gravid uterus	[10]	Yes	Yes	Yes	Yes	Yes	Yes	Yes	Yes	Included
Spontaneous rupture of the urinary bladder in the second trimester of pregnancy: a case report	[11]	Yes	Not clear	Yes	Yes	Yes	Yes	Yes	Yes	Included
Spontaneous combined bladder and uterine rupture in pregnancy	[12]	Yes	Yes	Yes	Yes	Yes	Not clear	Yes	Yes	Included
Spontaneous rupture of the bladder in pregnancy. A case report	[13]	Not clear	Yes	Yes	Yes	Yes	Yes	Yes	Yes	Included

In our analysis, the age range of women affected by SRUB during pregnancy was 27-39 years. Two were gravida (G) 4, two G2, and one G5. Four were in their second trimester of pregnancy [10-13], and one in her third [9]. Two had undergone previous cesarean sections [9,12], and one had an appendectomy and salpingectomy due to an ectopic pregnancy [13]. One case [12] reported a history of alcohol and drug abuse. Patients most commonly presented with abdominal pain [9-13], abdominal distension [9,11-13], oliguria and voiding difficulty [9,11,13], hematuria [10,12], fever [9,13], and vomiting [13]. Laboratory findings included elevated urea and creatinine levels in two cases [9,11,13], increased WBCs with a shift to neutrophilia in one case [13], and anemia and thrombocytopenia in the case of the combined uterus and bladder rupture [12]. The diagnosis of SRUB was primarily made by emergency laparotomy due to the patient's conditions [9-12]. Only in one case was an urgent ultrasound of the pelvis conducted, which showed a normal 16-week fetus with evidence of free fluid in the peritoneal cavity [13]. Besides its diagnostic role, laparotomy was the treatment method in all cases [9-13]. The size of the tear ranged from 2 to 6 cm [9-13]. Treatment protocols included tear repair, antibiotic therapy, and urinary catheterization. In all instances, the tear was repaired in two layers [9-13]. The duration of urinary catheterization spanned from 2 to 22 weeks in three studies [9-11] but was not specified in the other two [12,13]. After recovery and hospital discharge, four cases proceeded with uneventful pregnancies, culminating in the birth of full-term healthy babies [9-11,13]. One pregnancy was terminated due to a combined rupture of the uterus and bladder, necessitating a hysterectomy [12]. A summary of the included studies is presented in Table 2.

**Table 2:** Summary of case reports. G: Gravid; P: Parity; Ab: Abortion; C.S.: Cesarean section.

Study	Age	GPAb	Time of Rupture	Past Medical History	Signs and Symptoms	Lab Test	Method of Diagnosis	Findings in Laparotomy	Treatment	Prognosis
Shambe IH et al. (2015) [9]	30	G4P3	The third trimester of pregnancy	3 Previous C.S. Placenta acreta during her second pregnancy.	Generalized abdominal pain Viding difficulty Oliguria Abdominal tenderness Abdominal distension Fever	Elevated urea and creatinine	Abdominal ultrasound Laparotomy	Intraperitoneal bladder rupture peritoneal cavity walled off by omentum and contained 3.5 L of a clear yellow fluid A 6 cm tear in the dome of the urinary bladder with ragged edges Anteverted pregnant intact uterus	The repair of a tear in two layers with Vicryl 0 sutures Antibiotic therapy Catheterization for two weeks	Uneventful pregnancy Delivery by C.S. at full-term pregnancy
Graves J (1960) [10]	30	G2P1	The second trimester of pregnancy	One previous normal vaginal delivery No medical illness	Generalized abdominal pain Abdominal tenderness & rigidity Abdominal distension Hematuria	Not mentioned	Laparotomy	Intraperitoneal bladder rupture 2.5 liter of blood-stained urine in the abdominal cavity A 4-6 cm tear in the dome of the urinary bladder	The repair of a tear in two layers with Catgut 0 sutures Antibiotic therapy Catheterization for three weeks	Uneventful pregnancy Delivery by C.S. at full-term pregnancy
Faraj R et al., (2008) [11]	35	G4P3	The second trimester of pregnancy	Not mentioned	Generalized abdominal pain Viding difficulty	Elevated urea and creatinine	Laparotomy	Intraperitoneal bladder rupture 2.5 liter of clear yellow fluid in the peritoneal cavity 2 cm tear in the dome of the urinary bladder Retroverted pregnant intact uterus	The repair of a tear in two layers Antibiotic therapy Catheterization for the remainder of the pregnancy	Uneventful pregnancy Delivery by C.S. at full-term pregnancy
McCarthy FP et al. (2008) [12]	27	G5P4	The second trimester of pregnancy	Four previous CS A history of alcohol and intravenous drug abuse	Severe abdominal pain Vaginal bleeding	Anemia (Hb:9) Low platelet (Plt: 90,000)	Laparotomy	Bladder rupture in the posterior wall Uterus rupture Macerated fetus partly in the uterus and partly in the bladder Placenta acreta	The repair of a tear in two layers Hysterectomy Antibiotic therapy Urinary catheterization	Not mentioned
Shroff S et al. 1994 [13]	39	G3P1Ab1	The second trimester of pregnancy	Appendectomy Right tubectomy due to ectopic pregnancy	Generalized abdominal pain Voiding difficulty Oliguria Abdominal distension Fever Vomiting	Elevated urea Neutrophilia	Abdominal ultrasound Laparotomy	Intraperitoneal bladder rupture 800 cc of yellowish fluid in the peritoneal cavity Rupture of the fundus of the bladder, which admitted two fingers	The repair of a tear in two layers Peritoneal lavage with tetracycline, 1 g/1 of normal saline Antibiotic therapy Urinary catheterization	Uneventful pregnancy

Discussion

Pregnancy involves remarkable orchestration of physiologic changes. The kidneys grow in size during pregnancy. Dilatation of the urinary collecting system occurs in 80% of women and is related to smooth muscle relaxation caused by progesterone, relaxin, and mechanical obstruction from the gravid uterus. Up to 90% of pregnant women have physiological hydronephrosis, which is more common in the right kidney. Following voiding, detrusor muscle tone decreases, resulting in increased capacity and a higher residual urine volume [17]. These changes increase women's risk of urinary injuries in pregnancy and childbirth [18]. Although SRUB in pregnancy is uncommon, it can be fatal if not treated promptly. Identifying the manifestation, risk factors, method of diagnosis, treatment, and prognosis leads to more timely and integrated management. In our review, the cases of SRUB in pregnancy were intraperitoneal. The bladder dome was ruptured in four cases, and the posterior wall of the bladder was ruptured in one. According to the literature, bladder wall weakness is the primary cause of SRUB [19]. The urinary bladder is most vulnerable when distended and can rupture from the weakest point. The urine extravasation will be intraperitoneal if the bladder rupture is above the peritoneal reflection (on the bladder dome) and extraperitoneal if the bladder rupture is below the peritoneal reflection and not on the dome [20]. According to our review, the most common clinical signs were severe abdominal pain, distention, voiding difficulty, and hematuria. Other non-specific presentations of bladder ruptures included oliguria, fever, and vomiting. The classic triad of intraperitoneal rupture includes macroscopic hematuria, abdominal pain, and difficulty or inability to urinate. On the other hand, extraperitoneal bladder rupture is most commonly characterized by abdominal pain and dysuria [21]. Despite its low incidence and non-specific symptomatology, SRUB is frequently misdiagnosed. In a case reported by Faraj R et al. [11], the patient presented with abdominal pain and voiding difficulty three days before admission and was treated for a lower UTI. In another case reported by Shambe IH et al. [9], the patient had abdominal pain worsening for several days before admission and had also been diagnosed with a UTI. This review sheds light on the significance of having a high index of suspicion, timely diagnosis, and immediate surgical intervention in successful management and high-quality outcomes.

Lab test changes associated with SRUB are non-specific. For instance, elevated urea and creatinine levels were observed in three cases [9,11,13]. An increase in white blood cells with a shift to neutrophilia was noted in one case [13], while anemia and thrombocytopenia were seen in the case of the combined uterus and bladder rupture [12]. Biochemical markers are important to make an accurate diagnosis, evaluate the risk assessment, and affect the choice of treatment, which can, in turn, help to improve clinical outcomes. Urea and creatinine are reliable indicators of kidney health; increased serum levels suggest impaired kidney function [22]. While lab tests alone may not conclusively diagnose SRUB, they can be valuable when combined with a detailed history and thorough physical examination.

In every case reported, laparotomy was a diagnostic tool when radiological examinations failed to reveal a specific diagnosis [9-13]. Because almost all of the reported cases were diagnosed intraoperatively in a laparotomy for acute peritonitis, exploratory laparotomy is considered the gold standard of diagnosis. Following a literature review, we discovered that CT cystography is the best recommended preoperative evaluation for SRUB, as it allows simultaneous assessment of multiple abdominal organs [23]. However, it is contraindicated in pregnancy [24]. In the included cases, the treatment entailed the surgical repair of the rupture through a laparotomy procedure. There are no specific treatment guidelines for SRUB. According to the European Association of Urology, an intraperitoneal bladder rupture should always be treated with standard surgical repair because it can lead to a life-threatening condition due to the risk of abdominal sepsis and peritonitis. On the other hand, extraperitoneal bladder rupture can be treated conservatively [25,26]. In all five cases of our review, SRUB was treated with effused urine drainage, perforation site closure, intense antibiotic therapy, and urinary catheterization [9-13]. The bladder is one of the weakest organs concerning tissue strength. Despite its thin wall, the bladder is an incredibly resilient organ that recovers quickly from injury and subsequent repair. Although techniques vary greatly, bladder defects can be closed in one or two layers with a running non-locked stitch or interrupted sutures. Polyglactin or poliglecaprone absorbable sutures are the most commonly used for bladder repair because they cause minimal tissue reaction and dissolve in three weeks [27]. Plain catgut sutures cause even less bladder tissue reaction but last longer than polyglactin or poliglecaprone, which is unnecessary given the swift bladder tissue regeneration and recovery [25]. Sutures made of silk or Mersilene cause the most tissue reaction and are not recommended for bladder repair [28].

The duration of catheterization ranged from 2 to 22 weeks [9,11]. Surprisingly, the duration of catheterization following SRUB and subsequent urinary bladder repair has rarely been studied or reported, especially in the last 5-10 years. The lack of concrete evidence has led to practices based on expert opinions, which can be highly variable. Prolonged catheterization can impose clinical risks and burdens on patients' psychosocial status [29]. Any efforts toward an evidence-based approach to early catheter removal after the surgery can reduce the iatrogenic infection rates.

One of the objectives of this review was to identify any risk factors that could predict the risk of SRUB in pregnancy. From our findings, a history of previous surgeries, such as a cesarean section, appendectomy, or salpingectomy, was observed in patients with SRUB [9,11-13]. This could be explained by the increased risk of adhesion, which could weaken the bladder wall. Adhesion bands from previous abdominal surgeries can negatively affect the strength of the bladder wall by causing adhesions between the bladder and surrounding structures. These adhesions can create areas of weakness in the bladder wall and increase the risk of injury to the bladder. According to our review, placenta acreta was found in two cases [9,12]. In the first case, the patient had a history of placenta acreta in her second pregnancy. It is possible that the bladder wall was weakened during the surgery and ruptured spontaneously during the index pregnancy [9]. In another case, placenta acreta was discovered intraoperatively during a laparotomy, resulting in spontaneous uterus and bladder rupture [12].

Regarding past medical history, one patient among the cases had a history of alcohol and drug abuse. Alcohol intoxication has been reported as an etiology in a subset of cases of idiopathic bladder rupture [30]. Dooldeniya et al. associated episodes of SRUB (without concurrent bladder trauma) with either acute alcohol intoxication or recent significant alcohol consumption [31]. Patients intoxicated by alcohol exhibit altered sensorium, impaired consciousness, and abnormal behavioral reactions to bladder distension. These factors increase the likelihood of spontaneous bladder rupture and abnormal behavioral responses to bladder filling, increasing the SRUB risk [32]. An earlier report suggested that urinary retention in these cases could be due to rapid bladder-filling distention caused by alcohol's diuretic effect. Urinary bladder distention can result in an atonic decompensated bladder that becomes stretched and thinner [33]. Over-distension and thinning of the dome of the urinary bladder, which is the weakest point of the bladder anatomy, can eventually lead to spontaneous bladder rupture [34].

According to our review, the prognosis of SRUB in pregnancy in all cases was favorable. Prompt diagnosis and treatment of SRUB resulted in an optimal outcome. By presenting all the published evidence, we believe this review will draw significant attention from healthcare providers, especially obstetricians and midwives, to SRUB in pregnancy, despite its rarity. The primary limitation of this review is that the included studies are solely case reports. However, this can be attributed to the rarity of this pregnancy-related complication.

## Conclusions

In pregnancy, it is frequently difficult to distinguish SRUB from other causes of acute abdomen, particularly when there is no traumatic event history. SRUB frequently manifests with non-specific symptoms, resulting in missed or delayed diagnosis. The clinical presentation is usually with features of peritonitis, and most of the time, the definitive diagnosis is made intraoperatively. When patients present with urinary symptoms and abdominal pain suggestive of peritonitis, the possibility of bladder rupture should be considered, particularly in those who have had previous surgery such as a cesarean section. Early detection and treatment of SRUB are critical for an uneventful recovery and favorable outcome. Early urologic consultation and multidisciplinary approach management are critical for an optimal prognosis.
